# Mindfulness Is Related to the Situational Awareness of Medical Students Confronted with Life-Threatening Emergency Situations

**DOI:** 10.3390/jcm10091955

**Published:** 2021-05-02

**Authors:** Jacek Chmielewski, Kacper Łoś, Napoleon Waszkiewicz, Włodzimierz Łuczyński

**Affiliations:** 1 Department of Psychiatry, Medical University of Białystok, Plac Brodowicza 1, 16-070 Choroszcz, Poland; jacekchmielewski9@gmail.com (J.C.); napoleonwas@yahoo.com (N.W.); 2 Department of Medical Simulations, Medical University of Białystok, Szpitalna 30, 15-295 Białystok, Poland; kacperlos13@gmail.com

**Keywords:** stress, emergency medicine, mindfulness, situational awareness

## Abstract

Background: Emergency medicine can impose a heavy psychological burden on healthcare workers. Stress experienced during life-threatening situations may disrupt situational awareness (SA), i.e., the perception of environmental elements with respect to time and space, the comprehension of their meaning, and the projection of their state into the near future. We aimed to investigate whether mindfulness (a special way of paying attention: conscious, non-judgmental, and oriented to the present moment) can be related to the SA levels among final-year medical students confronted with life-threatening situations during medical simulations. Methods: The simulations were constructed as high-fidelity scenarios in children and adults (ClinicalTrials.gov ID: NCT03761355). The components of mindfulness were assessed using the Five Facet Mindfulness Questionnaire. SA among students was assessed using The Situation Awareness Global Assessment Technique at three levels: (1) data, (2) comprehension, and (3) projection. Results: In total, 117 students were included. Level 1 SA positively correlated with the overall mindfulness score and its components, i.e., nonreactivity, conscious presence, and nonjudgment. Moreover, level 3 SA significantly correlated with the description, but not with the overall mindfulness score. A regression model showed that nonreactivity explained 34% of Level 1 of SA variability. The addition of conscious presence and nonjudgment into this model did not change its predictive value. Conclusions: nonreactivity a component of mindfulness of final-year medical students is related to the meticulous data collection of patients in life-threatening situations.

## 1. Introduction

Emergency medicine can impose a heavy psychological burden on healthcare workers and is challenging to teach via modern medical education methods. In recent years, medical simulation has been used as a tool for the safe and effective training of medical students in managing life-threatening situations [1]. When playing out high-fidelity scenarios, participants experience stress similar to that in actual emergency situations [2]. Thus, medical simulation is ideal for studying team behavior when confronted with life-threatening conditions in patients. Simulation is particularly useful for assessing and teaching non-technical skills, i.e., so-called human factors, including team management, teamwork, communication, situational awareness (SA), decision-making, resource management, safety, and professionalism [3]. Among them, SA is one of the most important non-technical skills in medicine [4].

SA is defined as the perception of environmental elements with respect to time and space, the comprehension of their meaning, and the projection of their state into the near future [4]. SA may be affected by fixation or fixation error, i.e., the lack of reassessment or revision of a diagnosis or action when everything indicates that the revision should be conducted. Fixation is a statement, such as “this and only this”, “everything but this”, or “everything is alright”. Fixation errors result in adverse events in medicine. Therefore, full SA among emergency medical teams leads to avoidance of fixation errors and the correct diagnosis and treatment.

The assessment of SA is carried out at three levels: data collection, comprehension, and projection of future status [5]. The first level (i.e., data collection or perception) requires collecting information from various sources (including a patient and their companions), analyzing records, and ordering additional tests. The second level (comprehension) involves the synthesis, interpretation, and prioritization of data obtained in the first step to understand the patient’s current state (i.e., their diagnosis and treatment). The third level (projection) involves predicting possible scenarios and considering alternative actions and steps [5]. According to our assessments, SA is the lowest-rated non-technical skill of final-year medical students during simulations [6]. This phenomenon has been observed over the last three years, most likely due to the lack of appropriate prior training.

Stress experienced during actual or simulated life-threatening situations may disrupt SA. A modern method for reducing stress is to practice mindfulness. Mindfulness is a special way of paying attention: conscious, non-judgmental, and oriented to the present moment; it is about seeing things “as they are” “here and now” [7]. Among medical students, being mindful is associated with a reduction in stress, anxiety, and depressive symptoms, and an improvement in mood and empathy (review: [8]). Mindfulness training and yoga seem to effectively reduce stress and anxiety in healthcare workers, providing them with the ability to manage stressful work demands [9].

We previously demonstrated that mindfulness is associated with students’ SA during a high-fidelity simulation when assessed indirectly and subjectively at a general level using the Ottawa scale [6]. In the present study, we aimed to investigate whether mindfulness is related to the SA levels among final-year medical students confronted with life-threatening situations using direct and objective methods, and a simulation freeze technique. Our findings may aid the development of a prediction model and possible improvement of SA, thereby improving stress management and performance of emergency medical teams.

## 2. Materials and Methods

### 2.1. Participants

The subjects in this study were final-year medical students (ClinicalTrials.gov ID: NCT03761355). The research was conducted between October 2019 and February 2020 in the Department of Medical Simulations at the Medical University of Białystok, Poland. Only medical students who provided consent to participate in the study were included. Those who were pregnant or had participated in any form of meditation or mindfulness training prior to and during the study were excluded.

### 2.2. Simulations

The simulations were constructed as high-fidelity scenarios in life-threatening situations in children and adults, as previously described [6]. Each scenario had one technical and one non-technical goal. During the simulation scenario implementation, the students played different roles (team manager, member of the medical team, or actor—the patient’s caregiver). The group of students playing the role of doctors comprised five persons. The analysis concerns only the scenarios in which the students acted as team leaders.

### 2.3. Assessment of Mindfulness

Components of mindfulness were assessed in medical students prior to simulations using the short version of the Five Facet Mindfulness Questionnaire (FFMQ) in the Polish adapted and validated form [10,11]. FFMQ evaluates five factors: (1) conscious presence—acting with awareness of what one is doing in a given moment; (2) nonjudgmental observation of thoughts, emotions, and sensual impressions; (3) nonreactivity—low reactivity to stimulation, increased ability to focus, and widened perspective compared to normal experience; (4) description—the ability to view experiences with detachment, and to label them; and (5) observation—the ability to differentiate sensations deriving from muscle activity, inner organs, and parts of the body [10]. The higher the FFMQ score, the higher the level of mindfulness.

### 2.4. Assessment of Situational Awareness

During the patient’s life-threatening situation, SA among students was assessed using The Situation Awareness Global Assessment Technique (SAGAT), which was developed by Endsley [12]. After 10 min of simulation, the scenario was frozen, and students answered questions previously produced by a teacher relating to SA. Three levels of SA were measured: (1) Data, (2) Comprehension, and (3) Projection.

Critical points of each scenario were identified, and requirements (whose fulfillment resulted in 100% SA at a given level) were determined. Specialists in certain fields of medicine (e.g., a pediatric cardiologist) provided consultations about the points. We also consulted a psychologist about a method of stopping the scenario and the behavior during a freeze. The patient monitor was switched off during the freeze, and simulation participants turned their back on the patient to answer questions included in the questionnaire they had not seen before.

### 2.5. Ethics Approval

The study design was approved by the Ethics Committee at the Medical University of Bialystok in accordance with the Declaration of Helsinki (No R-I-002/358/2017). Signed informed consent was obtained from all students.

### 2.6. Data Presentation and Analysis

Data are presented as means and standard deviation (SD), and rates of incidence of a given characteristic in the evaluated group of students. Data were not normally distributed and non-parametric tests were used. Univariate analysis was conducted using the Mann–Whitney U test for continuous variables and the Chi-square test for the nominal ones. The Spearman’s rank correlation coefficient was used to evaluate the relationships between mindfulness and SA of medical students in the simulations. Multivariate linear regression was used to evaluate the impact of mindfulness and its components on students’ situational awareness during simulations. *p* values were corrected for multiple comparisons. Statistical analysis was performed using the Statistica 13 software (StatSoft, Tulsa, OK, USA). Only students with all data available were included in the analysis.

## 3. Results

In total, 180 final-year medical students attended compulsory (according to the study syllabus) simulations of life-threatening situations involving patients; among them, 65% agreed to participate in the study. The main reason for consent refusal was the lack of time to complete the survey. In total, 117 students (mean age of 24.5 ± 1.8 years) were included; 77 (65.8%) were female. Students who agreed to participate in the study did not differ in scores from those who did not provide consent in terms of sex, age, or technical and non-technical skills assessment. The average mindfulness scores of students from both overall and individual subscale assessments are presented in Table 1.

As each student played the role of a team leader in a pediatric emergency situation at least once, 117 simulations were analyzed. Table 2 shows an exemplary list of questions for the direct and objective assessment of SA in one of the scenarios. The average scores for the three SA levels achieved during the simulation scenario freeze are presented in Table 3. The students had significantly lower scores on level 3 SA than levels 1 and 2 (*p* < 0.01).

Then, correlations between mindfulness components and SA among students in the simulation scenarios of life-threatening situations were analyzed (Table 4). Level 1 SA positively and significantly correlated with the overall mindfulness score and some of its components, i.e., nonreactivity, conscious presence, and nonjudgment. Moreover, level 3 SA significantly correlated with the description, but not with the overall mindfulness score. No relationships were found between other components of mindfulness and students’ SA during the simulation.

A regression model was developed to assess the effect of the mindfulness and its specific components on students’ situational awareness at different levels. Level 1 of SA was significantly affected only by nonreactivity as a component of mindfulness. This model explained 34% of feature variability (*p* < 0.0001). The addition of conscious presence and nonjudgment into this model did not change its predictive value. The regression model for SA level 3 including description was not significant.

## 4. Discussion

Our study involving final-year medical students demonstrates that components of mindfulness are related to the students’ SA during life-threatening situations involving patients. These findings suggest the need for randomized trials with the use of mindfulness as an intervention to see if mindful team members will develop better SA, avoid fixation errors and improve the effectiveness of their actions.

Although SA is generally understood to mean “to know what is going on”, it is difficult to precisely define, describe, and understand this concept for both laymen and professionals [13]. In the last two decades, the assessment of SA (and attempts to improve it) have sparked an interest in studying human behavior in teams. However, only a limited number of studies have evaluated the influence of personality traits on SA [14]. Loss of SA often occurs in stressful and unexpected situations, like those in emergency medicine [14]. Yet, how to improve SA so that stress related to caring for patients in life-threatening situations does not affect doctors’ performance remains unclear [15]. In our opinion, this heightens the need to identify markers of SA and methods to improve SA among medical students.

In modern medicine, SA is the result of receiving information from multiple sources. Humans’ perceptions of reality are not always accurate in complex and constantly changing working conditions. This perception is related to cognitive, communication, and environmental factors, as well as team interactions. The maintenance of good SA is also influenced by nonhuman factors, including evolving medical technologies, which may lead to a doctor being distanced from the clinical situation. The human mind has limitations on processing information and, under certain circumstances, inattentional blindness may occur. In this context, SA can be seen as a product of individual perception, comprehension of available information, and projection of future events.

Objective assessment of SA in actual and simulated conditions is challenging. The direct and objective SAGAT method used in this study requires freezing (i.e., stopping the simulation scenario), which distracts students and may negatively impact the learning process [16]. Rosenman et al. developed a new three-level method for SA assessment, similar to SAGAT, but the assessment is carried out immediately after the simulation ends [5]. Hence, SA was correlated with clinical performance, but not with team perception of shared understanding, team leader effectiveness, and team experience. Nonetheless, the tool developed by Rosenman et al. could be a useful alternative to scenario freezes that are hard to perform using the SAGAT method. However, in our opinion, assessing level 1 and level 2 SA after the simulation is completed (i.e., when team members have more information than during the scenario) is troublesome. In addition, the scenario may last for different times in different teams, and thus, the achieved SA level may differ between teams.

In a study similar to ours, Coolen et al. used two or more freezes of the simulation scenario to assess the SA of pediatric team members [17]. According to the authors of this report there is a strong correlation between level 1 and level 2 SA (i.e., between the consensus on the primary problem during the first freeze and consensus on diagnosis during the second freeze) and quicker achievement of the scenarios’ goals. Meanwhile, our results showed that higher conscious presence improves SA for data collection and partly for the projection of future status, but not for a diagnosis.

Another important marker of SA is the team leader’s experience. It is widely believed that students and young doctors can reach a satisfactory first and second level of SA, but never third [14]. Likewise, our results indicate that students achieved the highest percentage of level 2 SA, followed by level 1 and then level 3 (<50%). One study demonstrated that the level of non-technical skills of young doctors mainly depends on their previous experience, i.e., the degree of residency [18]; however, we eliminated this element in our participants as only final-year students were included.

The relationship between personality traits such as mindfulness and the SA of healthcare professionals during life-threatening situations in patients has not been investigated so far. Awareness forms the basis of both characteristics, which encompasses awareness of self and surroundings. A previous study showed that mindfulness meditations led to an improvement in executive functioning and critical thinking; however, these results were not significantly better than those using a different type of meditation (sham meditation) [19]. Mindfulness training certainly improves such non-technical skills as communication with patients (review: [20]). It can also lead to an increase in the professionalism of students and young doctors, including awareness of their own limitations, determination of priorities, and seeking help when necessary [21]. A wide range of SA also includes these skills.

Our findings suggest that mindfulness is associated with the SA at the first level and partially at the third level. However, the regression analysis showed that only reactivity as one of the components of mindfulness can explain partially the variability of SA at the first level. Thus, future trials with intervention will show if an improvement in mindfulness will likely lead to an improvement in SA at levels 1 and 3. In clinical cases, level 1 SA involves gaining information from medical history, physical examination, and the results of additional tests. Although level 1 SA does not include the interpretation or integration of data, an improper action at this level may lead to the formation of an incorrect picture of the situation, and thus, to diagnostic and treatment errors (levels 2 and 3). On the other hand, level 2 SA skills (i.e., diagnosis and treatment) are learned (knowledge of standards) and may not be subject to personality traits such as mindfulness. Our study also shows that a component of mindfulness (such as description) could be related to the projection of patient outcome in life-threatening situations. It is possible that students’ open and clear minds during the simulation could lead to the better projection of their actions in the future. The role of specific mindfulness components in the awareness skills of medical students should be considered. In our opinion, from theoretical point of view such components like Describing, Nonjudge and Observe should be associated with Level 1, Observe also with Level 2 and Actaware with Level 3. Fino et al., 2021 found that nonjudgment of experience, acting with awareness and non -eacting were related to stress reduction in medical students [22]. However, they did not observe this effect with describing and observing. Our results can be partly explained by a study performed by Frere et al., which showed that level 1 (but not levels 2 and 3) SA was most frequently observed during Objective Structured Clinical Examination (OSCE) [23]. However, better tools are needed to assess levels 2 and 3 of SA to clarify the relationship between mindfulness and SA.

The introduction of mindfulness in emergency departments is feasible and may improve the well-being of healthcare professionals working in stressful conditions [24,25]. Focusing on the present moment can help both young doctors and their patients. It may also prove beneficial for dealing with stressful situations and effective medical education. Mindfulness certainly provides a tool to distance oneself from a stressful work environment, which can positively impact the objective assessment of the clinical situation. Indeed, the simulation room could become the place where medical students learn to react with awareness, recognize the space between stimulus and response, and consciously respond to stress stimuli [26].

The strengths of our study include the large number of simulations and the preparation of extensive questionnaires for SA assessment. Moreover, we performed objective and direct SA assessment using the SAGAT scale (contrary to subjective and indirect assessment, i.e., by instructors or after completing the scenario). The major limitation of this study is that SA was assessed during only one freeze. Besides, we only assessed the team leader’s SA, and the SA of other team members is likely different (usually lower), especially among those delegated to perform technical activities (monitoring, intubation, ECG, looking for medications, etc.). In addition, whether the students participating in the simulations had any mental disorders that would impede SA was unknown. Moreover, we did not investigate psychological interactions among team members.

Finally, although simulations are not actual stressful situations, our personal (and other authors’) experience suggests that students consider the simulations very realistic, and the stress that appears while playing the scenario is similar to that experienced in actual life-threatening situations. Nonetheless, further research is needed to understand the impact of SA on the performance of emergency teams in life-threatening situations. Further studies are also required to examine whether mindfulness training and the development of conscious presence influence SA and improve diagnostics and therapy.

## 5. Conclusions

In summary, the nonreacivity, a component of mindfulness, of final-year medical students is related to the meticulous collection of patient data in life-threatening situations. The impact of mindfulness training on improving SA among emergency teams and on actual patient care should be further investigated via randomized studies.

## Figures and Tables

**Table 1:** Results of the mindfulness assessment of final-year medical students.

Feature	Mean ± SD
Nonreact	2.77 ± 0.7
Observe	3.41 ± 0.8
Actaware	3.12 ± 0.4
Describe	3.40 ± 0.7
Nonjudge	3.10 ± 0.8
Total	3.16 ± 0.4

**Table 2:** An example of situational awareness assessment in a high-fidelity simulation scenario of a life-threatening situation involving a patient. The theme of the scenario was congenital metabolic disorder. The time from the start of the scenario to the freeze was 10 min.

**Level 1: Data** □ Did the baby receive the vaccine after birth? yes/no/don’t know □ Did the baby have normal weight gain after birth? yes/no/don’t know □ What was the patient’s systolic pressure at admission (mmHg)? 50–65/66–80/81–95/96–130/don’t know □ What was the patient’s blood sugar level at admission (mg%)? 30–45/46–60/61–75/76–100/don’t know □ What is the patient’s potassium level (mmol/L)? 2.0–3.5/3.6–5.0/5.1–6.5/>6.6/don’t know □ What is the patient’s pH? acidosis/alkalosis/don’t know
**Level 2: Comprehension** □ Which examination is the most important in establishing a diagnosis in the patient? culture tests/genetic testing/hormone tests/don’t know □ What is the likely cause of the patient’s symptoms? parental negligence/infection/diabetes/congenital metabolic disorder/don’t know
**Level 3: Projection** □ Which consultant do you need in the near future? neonatologist/geneticist/endocrinologist (diabetologist)/infectious disease specialist/don’t know □ Which of the following medications will be the most important in treating your patient? insulin/saline/hydrocortisone/fludrocortisone/don’t know □ Will the patient’s condition improve/change significantly within the next 30 min? yes/no/don’t know

**Table 3:** Results of the assessment of the three levels of situational awareness among medical students. The results are presented as a ratio of the maximum scores that can be achieved.

Level 1 Data (max 15 points)	10.2 ± 1.8, i.e., 68.0 ± 12%
Level 2 Comprehension (max 6 points)	4.9 ± 1.2, i.e., 82.6 ± 20%
Level 3 Projection (max 5 points)	2.5 ± 1.2, i.e., 50 ± 24%

**Table 4:** Results of the correlation analysis between components of mindfulness and situational awareness of medical students assessed during the life-threatening situation.

	Mindfulness (Total)	Nonreact	Observe	Actaware	Describe	Nonjudge
Level 1 Data	0.31 **	0.39 ***	−0.02	0.27 *	0.18	0.23 *
Level 2 Comprehension	−0.15	−0.06	−0.19	−0.20	−0.02	−0.00
Level 3 Projection	0.11	0.04	−0.08	0.11	0.24 *	0.13

* *p* < 0.05, ** *p* <0.01, *** *p* < 0.001.

## Data Availability

The raw datasets including the database (in Microsoft Access) used to support the findings of this study are available from the corresponding author upon request.
